# AI literacy mediates AI assisted diagnosis participation and critical thinking among medical students under supervision

**DOI:** 10.1038/s41746-026-02521-9

**Published:** 2026-03-14

**Authors:** Yang Xin, Deng Yan, Luo Shuren, Luo Minyang, Lu Liuheng

**Affiliations:** 1 Guangxi Orthopedic Hospital, Nanning, China; 2 Guangxi University, Nanning, China; 3 Guangxi Medical University, Nanning, China; 4 Health Commission of Guangxi Zhuang Autonomous Region, Nanning, China; 5 The Second Affiliated Hospital of Guangxi Medical University, Nanning, Guangxi China

**Keywords:** Education, Psychology, Psychology

## Abstract

Concerns that AI tools may erode diagnostic reasoning contrast with claims that AI can foster higher-order thinking. This longitudinal study followed 372 medical students across 12 months of supervised rotations using an AI-assisted diagnosis system. AI-assisted diagnosis participation, AI literacy and medical critical thinking were assessed at baseline, 6 months and 12 months. Cross-lagged panel models examined prospective associations, statistical mediation by AI literacy and moderation by prior technological experience and learning goal orientation. Higher participation was associated with increases in AI literacy and critical thinking, and AI literacy statistically mediated the participation-to-critical thinking association. Indirect effects were stronger among students with greater technological experience and mastery-oriented goals and weaker among performance-oriented peers. Findings indicate that, within supervised clinical training, engagement with AI systems is associated with critical thinking development partly through enhanced AI literacy, supporting AI tools as educational resources under faculty guidance.

## Introduction

The integration of artificial intelligence (AI) into clinical workflows has transitioned from theoretical potential to daily practice, fundamentally altering the educational landscape for medical trainees^1–3^. Medical students in clinical rotations increasingly engage with AI-driven diagnostic support systems, ranging from radiology interpretation algorithms to large language models (LLMs) assisting in differential diagnosis^4^. Unlike traditional passive learning tools, these systems function as advanced probabilistic support tools, offering diagnostic probabilities and reasoning pathways that students must actively evaluate^5,6^. As AI becomes ubiquitous in medical training, a critical pedagogical question emerges: does frequent participation in AI-assisted diagnosis enhance medical students’ critical thinking capabilities, or does it lead to cognitive offloading and dependency?

Recent research presents a divided perspective on this cognitive impact. One strand of literature warns of “cognitive atrophy,” suggesting that over-reliance on automated diagnostic outputs may erode foundational reasoning skills and foster passivity^7,8^. Conversely, proponents argue that AI tools can serve as “cognitive scaffolds,” handling routine information processing and freeing cognitive resources for higher-order analysis and synthesis^9^. However, much of the existing evidence relies on cross-sectional surveys or experimental snapshots, failing to capture the developmental trajectory of cognitive skills over time^10^. Consequently, a substantive gap remains in understanding the longitudinal mechanisms through which sustained interaction with AI systems influences the maturation of critical thinking during clinical training.

To address this gap, the present study frames the relationship between AI usage and critical thinking through the lens of Situated Learning Theory. We posit that “AI-Assisted Diagnosis Participation” within a supervised hierarchical medical team represents not merely a background variable but a form of legitimate peripheral participation—an active learning behaviour embedded in authentic clinical practice. Within this supervisory structure, guidance from senior physicians provides the necessary pedagogical scaffolding for students to interrogate AI outputs without compromising patient safety. Through repeated, situated engagement with AI tools—a process of “learning by doing”—students progressively acquire specific technical and cognitive competencies, herein conceptualised as “AI Literacy”^3,11^. Crucially, we distinguish this construct from mere usage frequency; AI literacy represents the capacity to comprehend algorithmic logic, evaluate probabilistic outputs, and effectively integrate AI recommendations into clinical reasoning^12^. Accordingly, we hypothesise that participation serves as the behavioural antecedent that fosters the developmental acquisition of this sophisticated literacy over time.

Furthermore, this study proposes that AI literacy acts as the essential cognitive bridge to critical thinking development. Medical critical thinking involves the disciplined evaluation of evidence, identification of biases, and synthesis of conflicting data^13,14^. We argue that students with high AI literacy possess the requisite “algorithmic awareness” to interrogate AI-generated suggestions rather than accepting them blindly. This evaluative process—identifying algorithmic hallucinations, recognizing data biases, and reconciling AI outputs with clinical context—constitutes a rigorous exercise in critical reasoning^15,16^. Thus, AI literacy theoretically mediates the relationship between participation and critical thinking: active usage builds the competence (literacy) necessary to engage in the high-level evaluation (critical thinking) that prevents cognitive dependence.

Recognizing that this developmental process is not uniform, the analyses also incorporate individual difference factors based on Achievement Goal Theory^17^. We recognize that the developmental pathways linking AI-assisted diagnosis participation, AI literacy, and medical critical thinking are likely to vary across learners^5^. Prior technological experience may shape how comfortable students feel experimenting with AI tools, while learning goal orientations may influence whether they approach AI-assisted tasks as opportunities to deepen understanding or primarily as means to secure correct answers^18–20^. Mastery-oriented students, who prioritize learning and competence development, may be more inclined to use AI tools for exploration and reflection, whereas performance-oriented students may focus on efficiency and error avoidance, potentially reinforcing shortcut-based use of AI recommendations^19,21^. Students’ orientation toward mastery (learning for competence) versus performance (learning for validation), alongside their prior technological experience, likely moderates the efficiency of these pathways^18,20,22^.

In the context of rapidly expanding AI deployment in health systems, AI literacy is increasingly regarded as a prerequisite for safe and accountable digital health practice, with emerging evidence that targeted educational interventions can improve learners’ understanding of AI concepts and their confidence in questioning algorithmic outputs^3,15^. Building on this work, the present study uses a three-wave longitudinal design across a 12-month clinical rotation to examine temporal associations among AI-assisted diagnosis participation, AI literacy, and medical critical thinking. Specifically, it investigates whether AI-assisted diagnosis participation is prospectively associated with subsequent critical thinking, whether AI literacy statistically mediates this association, and how these longitudinal pathways differ by students’ prior technological experience and learning goal orientations. By doing so, the study aims to provide timely empirical guidance on how AI augmented curricula and clinical learning environments can be structured so that routine use of AI supports, rather than undermines, medical students’ critical appraisal and diagnostic responsibility.

Critically, this study was conducted within a supervised clinical training context where experienced faculty mentors guided students’ interactions with AI diagnostic tools. This supervisory structure is theorised to be essential for the hypothesised pathway from AI participation to literacy to critical thinking. Without close mentorship, learners—who are not yet experienced practitioners—may struggle to discern whether discrepancies between their own clinical judgment and AI-generated outputs reflect their own diagnostic errors or algorithmic limitations^6^. The presence of expert guidance creates a pedagogically “safe” learning environment where students can experiment with AI tools, receive immediate corrective feedback, and iteratively develop the critical evaluation skills that constitute AI literacy^23^. Thus, while we hypothesise that AI-assisted diagnosis participation fosters AI literacy, we emphasise that this developmental process is unlikely to occur through passive or unsupervised exposure to AI systems. Throughout this manuscript, “AI-assisted diagnosis participation” refers specifically to engagement with AI tools under structured faculty supervision during clinical rotations.

## Results

### Preliminary analyses and measurement validity

Analyses were based on the 372 students who completed all three measurement waves. Sample characteristics and attrition comparisons are summarised in Table 5. There were no statistically significant differences between the baseline and retained samples in age, gender, clinical year, cumulative GPA, prior technological experience, or learning goal orientations, and effect sizes for these comparisons were small (all |d|≤ 0.05; Table 5).

Descriptive statistics and bivariate correlations for the primary study variables are presented in Table 1. Mean AI-assisted diagnosis participation increased over time, from 3.12 (SD = 1.18) at baseline to 4.67 (SD = 1.09) at six months and 5.41 (SD = 0.98) at 12 months. AI literacy showed a similar pattern, with means of 3.45 (SD = 0.87), 4.89 (SD = 0.79), and 5.67 (SD = 0.73) at T₀, T₁, and T₂, respectively. Medical critical thinking scores increased more modestly, from 4.23 (SD = 0.68) at baseline to 4.52 (SD = 0.65) at six months and 4.78 (SD = 0.64) at 12 months. Skewness values ranged from −0.45 to 0.38 and kurtosis values from −0.52 to 0.41, suggesting that deviations from normality were limited and that the use of maximum likelihood estimation was appropriate.

**Table 1:** Bivariate correlations among primary variables (*N* = 372)

Variable	1	2	3	4	5	6	7	8	9	10	11	12
1. AI participation T₀	—											
2. AI participation T₁	0.64**	—										
3. AI participation T₂	0.58**	0.71**	—									
4. AI literacy T₀	0.49**	0.41**	0.38**	—								
5. AI literacy T₁	0.52**	0.68**	0.61**	0.59**	—							
6. AI literacy T₂	0.47**	0.63**	0.74**	0.54**	0.73**	—						
7. Critical thinking T₀	0.25**	0.28**	0.31**	0.54**	0.46**	0.43**	—					
8. Critical thinking T₁	0.27**	0.35**	0.38**	0.48**	0.58**	0.55**	0.68**	—				
9. Critical thinking T₂	0.29**	0.33**	0.42**	0.45**	0.54**	0.67**	0.61**	0.74**	—			
10. Prior tech experience	0.41**	0.35**	0.32**	0.43**	0.38**	0.35**	0.31**	0.29**	0.27**	—		
11. Mastery orientation	0.33**	0.36**	0.39**	0.37**	0.41**	0.44**	0.46**	0.48**	0.51**	0.24**	—	
12. Performance orientation	0.16**	0.09	0.05	0.14**	0.08	0.03	−0.06	−0.11*	−0.15**	0.13*	−0.21**	—
Descriptive statistics												
M	3.12	4.67	5.41	3.45	4.89	5.67	4.23	4.52	4.78	4.31	5.78	4.12
SD	1.18	1.09	0.98	0.87	0.79	0.73	0.68	0.65	0.64	1.22	0.91	1.43

*N* = 372 (complete cases analysis).

**p < 0.01; *p < 0.05.

Zero-order correlations showed substantial longitudinal stability within each construct (Table 1). For AI-assisted diagnosis participation, correlations across waves ranged from *r* = 0.58 to 0.71; for AI literacy, from *r* = 0.54 to 0.73; and for medical critical thinking, from *r* = 0.61 to 0.74. Correlations between AI-assisted diagnosis participation and AI literacy were moderate to large (*r* = 0.38–0.74), and correlations between AI-assisted diagnosis participation and critical thinking were smaller but consistently positive (*r* = 0.25–0.42). AI literacy was moderately associated with critical thinking at each wave (*r* = 0.43–0.67). Prior technological experience was positively related to AI participation, AI literacy, and critical thinking (*r* = 0.27–0.43), whereas performance orientation showed weak and partly negative correlations with critical thinking at later waves (e.g., *r* = −0.11 at T₁ and *r* = −0.15 at T₂; Table 1).

To examine whether AI-assisted diagnosis participation and AI literacy represented empirically distinct constructs, confirmatory factor analyses compared a two-factor solution (separating participation and literacy) with a more restrictive one-factor solution. The two-factor model showed clearly superior fit indices and lower information criteria than the one-factor model, supporting the conceptualisation of AI-assisted diagnosis participation as a behavioural construct and AI literacy as a competence construct.

Longitudinal measurement invariance testing supported configural, metric, and scalar invariance for AI-assisted diagnosis participation, AI literacy, and medical critical thinking across the three waves. These results indicate that the factor structures and response scales were stable over time and that comparisons of observed and latent means across waves are interpretable in terms of change rather than measurement artefacts.

### Longitudinal relationships: cross-lagged panel analysis

A three-wave cross-lagged panel model (CLPM) was used to examine temporal associations among AI-assisted diagnosis participation, AI literacy, and medical critical thinking while controlling for baseline covariates (age, gender, and GPA). The model showed acceptable global fit to the data (χ² = 167.4, df = 89, RMSEA = 0.049, SRMR = 0.041).

Autoregressive paths indicated high stability for all constructs across adjacent waves (Table 2). AI-assisted diagnosis participation at T₀ predicted participation at T₁ (β = 0.640, SE = 0.039, *p* < 0.001, 95% CI [0.564, 0.716]) and participation at T₁ predicted participation at T₂ (β = 0.680, SE = 0.036, *p* < 0.001, 95% CI [0.610, 0.750]). AI literacy showed comparable stability from T₀ to T₁ (β = 0.590, SE = 0.042, *p* < 0.001, 95% CI [0.508, 0.672]) and from T₁ to T₂ (β = 0.610, SE = 0.038, *p* < 0.001, 95% CI [0.536, 0.684]). Medical critical thinking was also highly stable (T₀ → T₁: β = 0.710, SE = 0.033, *p* < 0.001, 95% CI [0.646, 0.774]; T₁ → T₂: β = 0.740, SE = 0.031, *p* < 0.001, 95% CI [0.680, 0.800]).

**Table 2:** Cross-lagged panel analysis: temporal relationships (*N* = 372)

Pathway	β	SE	*t*	*p*	95% CI
Autoregressive effects					
AI participation T₀ → AI participation T₁	0.640	0.039	16.41	<0.001	[0.564, 0.716]
AI participation T₁ → AI participation T₂	0.680	0.036	18.89	<0.001	[0.610, 0.750]
AI literacy T₀ →AI literacy T₁	0.590	0.042	14.05	<0.001	[0.508, 0.672]
AI literacy T₁ →AI literacy T₂	0.610	0.038	16.05	<0.001	[0.536, 0.684]
Critical thinking T₀ → critical thinking T₁	0.710	0.033	21.52	<0.001	[0.646, 0.774]
Critical thinking T₁ → critical thinking T₂	0.740	0.031	23.87	<0.001	[0.680, 0.800]
Cross-lagged effects (theoretical model)					
AI participation T₀ →AI literacy T₁	0.341	0.045	7.56	<0.001	[0.252, 0.428]
AI participation T₁ →AI literacy T₂	0.290	0.041	7.07	<0.001	[0.210, 0.370]
AI literacy T₀ → critical thinking T₁	0.160	0.046	3.48	<0.001	[0.070, 0.250]
AI literacy T₁ → critical thinking T₂	0.289	0.035	8.29	<0.001	[0.221, 0.359]
AI participation T₀ → critical thinking T₁	0.234	0.042	5.57	<0.001	[0.152, 0.316]
AI participation T₁ → critical thinking T₂	0.218	0.039	5.59	<0.001	[0.142, 0.294]
AI participation T₀ → critical thinking T₂ (Total)	0.237	0.041	5.78	<0.001	[0.157, 0.317]
Cross-lagged effects (alternative model)					
Critical thinking T₀ →AI literacy T₁	0.089	0.044	2.02	0.044	[0.003, 0.175]
Critical thinking T₁ →AI literacy T₂	0.072	0.041	1.76	0.079	[−0.008, 0.152]
AI literacy T₀ → AI participation T₁	0.127	0.047	2.70	0.007	[0.035, 0.219]
AI literacy T₁ → AI participation T₂	0.105	0.042	2.50	0.012	[0.023, 0.187]
Critical thinking T₀ → AI participation T₁	0.089	0.043	2.07	0.038	[0.005, 0.173]
Critical thinking T₁ → AI participation T₂	0.076	0.041	1.85	0.065	[−0.005, 0.157]
Within-time correlations					
AI participation ↔ AI literacy (T₀)	0.490	0.041	11.95	<0.001	[0.410, 0.570]
AI participation ↔ critical thinking (T₀)	0.250	0.048	5.21	<0.001	[0.156, 0.344]
AI literacy ↔ critical thinking (T₀)	0.540	0.038	14.21	<0.001	[0.466, 0.614]
AI participation ↔ AI literacy (T₁)	0.680	0.034	20.00	<0.001	[0.614, 0.746]
AI participation ↔ critical thinking (T₁)	0.350	0.045	7.78	<0.001	[0.262, 0.438]
AI literacy ↔ critical thinking (T₁)	0.580	0.037	15.68	<0.001	[0.508, 0.652]
AI participation ↔ AI literacy (T₂)	0.740	0.032	23.13	<0.001	[0.678, 0.802]
AI participation ↔ critical thinking (T₂)	0.420	0.043	9.77	<0.001	[0.336, 0.504]
AI literacy ↔ critical thinking (T₂)	0.670	0.035	19.14	<0.001	[0.602, 0.738]
Model fit indices					
Theoretical model					
χ²(df)	167.4(89)	—	—	<0.001	—
CFI	0.951	—	—	—	—
TLI	0.943	—	—	—	—
RMSEA [90% CI]	0.049 [0.037, 0.061]	—	—	—	—
SRMR	0.041	—	—	—	—
Alternative model					
χ²(df)	199.1(89)	—	—	<0.001	—
CFI	0.934	—	—	—	—
TLI	0.921	—	—	—	—
RMSEA [90% CI]	0.058 [0.046, 0.070]	—	—	—	—
SRMR	0.056	—	—	—	—
Model comparison					
Δχ²(df)	31.7(0)	—	—	<0.001	—
ΔAIC	31.7	—	—	—	—

Cross-lagged paths are summarised in Table 2. Higher AI-assisted diagnosis participation at baseline was associated with higher AI literacy at six months (β = 0.341, SE = 0.045, *p* < 0.001, 95% CI [0.252, 0.428]) and higher AI literacy at six months was associated with higher critical thinking at 12 months (β = 0.289, SE = 0.035, *p* < 0.001, 95% CI [0.221, 0.359]). Baseline AI-assisted diagnosis participation also predicted higher critical thinking at six months (β = 0.234, SE = 0.042, *p* < 0.001, 95% CI [0.152, 0.316]). A similar pattern was observed for the second interval: AI-assisted diagnosis participation at T₁ predicted AI literacy at T₂ (β = 0.290, SE = 0.041, *p* < 0.001, 95% CI [0.210, 0.370]) and critical thinking at T₂ (β = 0.218, SE = 0.039, *p* < 0.001, 95% CI [0.142, 0.294]). In addition, AI literacy at T₀ predicted critical thinking at T₁ (β = 0.160, SE = 0.046, *p* < 0.001, 95% CI [0.070, 0.250]), indicating that higher initial literacy was associated with subsequent increases in critical thinking.

Although cross-lagged panel models cannot definitively establish causal direction^24,25^, the longitudinal design permits examination of temporal precedence consistent with our theoretical framework. To assess whether the observed pattern of associations was equally consistent with an alternative temporal ordering, we specified a “reverse causation” model (i.e., Critical Thinking → AI Literacy → AI Participation). we conducted a sensitivity analysis to evaluate whether the observed longitudinal pattern was equally compatible with a reversed temporal ordering. Specifically, we estimated an alternative model in which baseline critical thinking predicted AI literacy at six months, which in turn predicted AI-assisted diagnosis participation at 12 months. Relative to the prespecified model, the alternative specification showed poorer fit based on the model comparison test reported in Table 2 (χ² difference = 31.7, *p* < 0.001) and a higher information criterion (ΔAIC = 31.7). In addition, the key reversed cross-lagged paths were small and less consistent (critical thinking T0 → AI literacy T1: β = 0.089, SE = 0.043, *p* = 0.038; AI literacy T1 → AI participation T2: β = 0.076, SE = 0.041, *p* = 0.065). Taken together, these results indicate that the data are more consistent with the hypothesised ordering than with the fully reversed specification; however, unmeasured confounding and alternative causal processes cannot be ruled out in this observational design. Importantly, the interpretation of directional associations rests primarily on the theoretical framework derived from Situated Learning Theory and the time-ordered measurement design, rather than on model fit comparisons alone.

The total effect of AI participation on critical thinking development across the complete temporal sequence achieved substantial magnitude (β = .237, SE = 0.041, *p* < 0.001), indicating that a one-standard-deviation increase in AI participation corresponds to a 0.237 standard deviation enhancement in critical thinking competency. This effect size exceeds typical technology-learning relationships documented in educational research while demonstrating practical significance within medical education contexts^26^.

### Longitudinal mediation analysis

To examine whether the longitudinal association between AI-assisted diagnosis participation and medical critical thinking was statistically mediated by AI literacy, we specified a three-wave mediation model focusing on the T₀ → T₁ → T₂ temporal sequence (Table 3). Specifically, this model tested whether baseline AI-assisted diagnosis participation was associated with AI literacy at six months, and whether AI literacy at six months was in turn associated with critical thinking at 12 months, after controlling for prior levels of all constructs. In this model, baseline AI-assisted diagnosis participation retained a modest direct association with critical thinking at 12 months after inclusion of the mediator pathway (direct β = 0.095, SE = 0.039, *p* = 0.015, 95% CI [0.018, 0.172]). Baseline AI-assisted diagnosis participation was positively related to AI literacy at six months (β = 0.341, SE = 0.038, *p* < 0.001, 95% CI [0.267, 0.415]), and AI literacy at six months was positively related to critical thinking at 12 months (β = 0.289, SE = 0.035, *p* < 0.001, 95% CI [0.220, 0.358]).

**Table 3:** Mediation analysis: AI literacy as mediator (*N* = 372)

Effect type and pathway	Estimate	SE	*p*	95% CI
Direct effects				
AI participation T₀ → critical thinking T₂	0.095	0.039	0.015	[0.018, 0.172]
AI participation T₀ →AI literacy T₁	0.341	0.038	<0.001	[0.267, 0.415]
AI literacy T₁ → critical thinking T₂	0.289	0.035	<0.001	[0.220, 0.358]
Indirect effects (Monte Carlo CI)				
AI part. T₀ →AI Lit. T₁ → crit. think. T₂	0.142	0.019	<0.001	[0.089, 0.201]
Total effects				
AI participation T₀ → critical thinking T₂	0.237	0.041	<0.001	[0.156, 0.318]
Mediation assessment				
Proportion mediated	0.38	0.07	—	[0.26, 0.54]
Sobel test	z = 5.74, *p* < 0.001	—	—	—
Alternative bootstrap methods				
Bias-corrected bootstrap (10,000 samples)	—	—	—	[0.091, 0.203]
Percentile bootstrap (10,000 samples)	—	—	—	[0.087, 0.198]
Model comparisons				
Partial mediation model				
χ²(df)	145.3(88)	—	<0.001	—
AIC	15,623.7	—	—	—
CFI	0.951	—	—	—
RMSEA	0.043	—	—	—
Complete mediation model				
χ²(df)	154.0(89)	—	<0.001	—
AIC	15,630.4	—	—	—
CFI	0.947	—	—	—
RMSEA	0.045	—	—	—
No mediation model				
χ²(df)	178.6(90)	—	<0.001	—
AIC	15,652.9	—	—	—
CFI	0.938	—	—	—
RMSEA	0.051	—	—	—
Model comparison tests				
Partial vs. complete mediation Δχ²(1) = 8.7, *p* = 0.003		—	—	—
Partial vs. no mediation χ²(2) = 33.3, *p* < 0.001		—	—	—

The indirect pathway was statistically significant: baseline AI-assisted diagnosis participation was associated with higher AI literacy at six months (β = 0.341, SE = 0.038, *p* < 0.001), and AI literacy at six months was associated with higher critical thinking at 12 months (β = 0.289, SE = 0.035, *p* < 0.001), yielding an indirect effect of β = 0.142 (SE = 0.019, *p* < 0.001, 95% CI [0.089, 0.201]). The total association between baseline AI-assisted diagnosis participation and critical thinking at 12 months was β = 0.237 (SE = 0.041, *p* < 0.001, 95% CI [0.156, 0.318]). Approximately 38% of this total association (95% CI [0.26, 0.54]) was transmitted through the sequential pathway involving AI literacy, indicating partial mediation.

This mediation proportion represents a moderate-to-large effect within educational technology research contexts, where indirect effects typically account for 20–35% of total associations^27^. Approximately 38% of the total longitudinal association between baseline AI-assisted diagnosis participation and critical thinking at 12 months (95% CI [0.26, 0.54]) was transmitted through AI literacy at six months, indicating partial mediation. This proportion suggests that AI literacy accounts for a meaningful share of the prospective association, while a statistically significant direct path remained (β = 0.095, SE = 0.039, *p* = 0.015), implying that additional pathways may also link participation with later critical thinking. The magnitude suggests that the sequential pathway from AI participation through AI literacy to critical thinking constitutes a substantial portion of the overall longitudinal association. That is, students who participated more actively in AI-assisted diagnosis tended to show higher AI literacy at the subsequent time point, and this higher literacy was in turn associated with higher critical thinking at the following time point. Because mediation estimates in observational longitudinal data remain vulnerable to unmeasured confounding, these proportions should be interpreted as describing statistical decomposition of associations rather than definitive mechanistic attribution. The remaining direct association indicates that participation may also relate to critical thinking through mechanisms not captured by AI literacy, warranting further investigation.

Monte Carlo confidence interval procedures with 10,000 resamples confirmed mediation effect stability across alternative estimation methods. Bias-corrected accelerated intervals (95% CI [0.091, 0.203]) and percentile intervals (95% CI [0.087, 0.198]) yielded similar ranges, supporting confidence in indirect effect estimates. Sensitivity analysis through varying distributional assumptions and missing data treatments produced consistent results, indicating robustness across analytical choices.

The partial mediation model demonstrated superior fit compared to complete mediation alternatives (Δχ² = 8.7, df = 1, *p* = 0.003), indicating meaningful direct effects alongside indirect pathways. The direct effect of AI participation on critical thinking remained significant (β = 0.095, SE = 0.039, *p* = 0.015) after accounting for AI literacy mediation, suggesting that additional mechanisms beyond AI literacy contribute to AI participation effectiveness for critical thinking development.

### Moderation by individual differences

Multi-group structural equation models were used to examine whether the longitudinal associations via AI literacy differed by prior technological experience and learning goal orientation (Table 4). The model revealed a “Matthew Effect.” For prior technological experience, the sample was split at the median into lower-experience (*n* = 186) and higher-experience (*n* = 186) groups. In the lower-experience group, the path from AI-assisted diagnosis participation to AI literacy was β = 0.287 (SE = 0.049, *p* < 0.001), the path from AI literacy to critical thinking was β = 0.271 (SE = 0.054, *p* < 0.001), and the indirect effect via AI literacy was β = 0.096 (SE = 0.029, *p* < 0.001, 95% CI [0.048, 0.155]). In the higher-experience group, the corresponding paths were somewhat larger: AI-assisted diagnosis participation to AI literacy β = 0.389 (SE = 0.045, *p* < 0.001), AI literacy to critical thinking β = 0.324 (SE = 0.048, *p* < 0.001), and the indirect effect β = 0.178 (SE = 0.031, *p* < 0.001, 95% CI [0.121, 0.245]). The direct path from AI-assisted diagnosis participation to critical thinking was small in both groups (β = 0.108, SE = 0.051, *p* = 0.034 in the low-experience group; β = 0.089, SE = 0.047, *p* = 0.060 in the high-experience group). A χ² difference test comparing a constrained model (χ²(156) = 198.7, *p* = 0.008) with an unconstrained model (χ²(152) = 186.3, *p* = 0.024) indicated that allowing the key structural paths to vary by technological experience improved model fit (Δχ²(4) = 12.4, *p* = 0.015). Supplementary continuous-variable moderation analyses using latent moderated structural equations corroborated these patterns: the interaction between AI participation and technological experience significantly predicted AI literacy, indicating that the association between participation and literacy was stronger at higher levels of technological experience (see Supplementary Table S3).

**Table 4:** Individual difference moderation analysis (*N* = 372)

Moderator configuration	Low experience (*n* = 186)	High experience (*n* = 186)	Low mastery (*n* = 185)	High mastery (*n* = 187)
Prior technological experience moderation				
Direct effects				
AI participation →AI literacy	0.287 (0.049)**	0.389 (0.045)**	—	—
AI literacy → critical thinking	0.271 (0.054)**	0.324 (0.048)**	—	—
AI participation → critical thinking	0.108 (0.051)*	0.089 (0.047)	—	—
Indirect effect				
Mediation pathway	0.096 (0.029)**	0.178 (0.031)**	—	—
95% confidence interval	[0.048, 0.155]	[0.121, 0.245]	—	—
Learning goal orientation moderation				
Direct effects				
AI participation →AI literacy	—	—	0.298 (0.051)**	0.412 (0.043)**
AI literacy → critical thinking	—	—	0.247 (0.058)**	0.354 (0.046)**
AI participation → critical thinking	—	—	0.134 (0.053)*	0.067 (0.045)
Indirect effect				
Mediation pathway	—	—	0.081 (0.023)**	0.189 (0.027)**
95% confidence interval	—	—	[0.032, 0.139]	[0.134, 0.256]
Combined moderation analysis				
Low experience × low mastery	0.052 (0.019)**	95% CI [0.018, 0.095]		
Low experience × high mastery	0.139 (0.026)**	95% CI [0.091, 0.189]		
High experience × low mastery	0.125 (0.024)**	95% CI [0.084, 0.174]		
High experience × high mastery	0.234 (0.037)**	95% CI [0.168, 0.312]		
Multi-group comparisons				
Prior experience				
Constrained model	χ²(156) = 198.7, *p* = 0.008			
Unconstrained model	χ²(152) = 186.3, *p* = 0.024			
Difference test	Δχ²(4) = 12.4, *p* = 0.015			
Goal orientation				
Constrained model	χ²(156) = 203.4, *p* = 0.005			
Unconstrained model	χ²(152) = 187.7, *p* = 0.021			
Difference test	Δχ²(4) = 15.7, *p* = 0.003			
Combined moderation				
Constrained model	χ²(164) = 234.8, *p* < 0.001			
Unconstrained model	χ²(152) = 201.3, *p* = 0.003			
Difference test	Δχ²(12) = 33.5, *p* = 0.001			

Unstandardized coefficients with standard errors in parentheses. Groups divided by median split procedures for Prior Technological Experience (Mdn = 4.25) and Learning Goal Orientation composite scores (Mastery - Performance orientation difference scores, Mdn = 1.42). Supplementary continuous-variable moderation analyses using latent moderated structural equations (LMS) are reported in Supplementary Table S3; these analyses yielded convergent findings.

**p < 0.01; *p < 0.05.

For learning goal orientation, students were divided into lower- and higher-mastery groups using a median split on mastery orientation scores (*n* = 185 and *n* = 187, respectively). In the lower-mastery group, AI-assisted diagnosis participation was associated with AI literacy (β = 0.298, SE = 0.051, *p* < 0.001) and AI literacy was associated with critical thinking (β = 0.247, SE = 0.058, *p* < 0.001), yielding an indirect effect of β = 0.081 (SE = 0.023, *p* < 0.001, 95% CI [0.032, 0.139]). The direct path from AI-assisted diagnosis participation to critical thinking was β = 0.134 (SE = 0.053, *p* = 0.012). In the higher-mastery group, the path from AI-assisted diagnosis participation to AI literacy was β = 0.412 (SE = 0.043, *p* < 0.001), the path from AI literacy to critical thinking was β = 0.354 (SE = 0.046, *p* < 0.001), and the indirect effect via AI literacy was β = 0.189 (SE = 0.027, *p* < 0.001, 95% CI [0.134, 0.256]). The direct path from AI-assisted diagnosis participation to critical thinking was smaller and not statistically significant in the higher-mastery group (β = 0.067, SE = 0.045, *p* = 0.137). A multi-group comparison showed that a model allowing these paths to differ by mastery orientation (χ²(152) = 187.7, *p* = 0.021) fit better than a constrained model (χ²(156) = 203.4, *p* = 0.005; Δχ²(4) = 15.7, *p* = 0.003). Continuous-variable moderation analyses yielded convergent findings: the AI Literacy × Mastery Orientation interaction significantly predicted critical thinking, confirming that higher mastery orientation strengthened the association between AI literacy and critical thinking outcomes. Full results from simple slope analyses and conditional indirect effects are presented in Supplementary Table S3.

To examine combined moderation, students were initially classified into four groups defined by the intersection of prior technological experience (low vs high) and mastery orientation (low vs high) using median splits. The estimated indirect effect from AI-assisted diagnosis participation to critical thinking via AI literacy varied across these groups (Table 4). In the low-experience/low-mastery group, the indirect effect was β = 0.052 (SE = 0.019, *p* = 0.005, 95% CI [0.018, 0.095]); in the low-experience/high-mastery group, β = 0.139 (SE = 0.026, *p* < 0.001, 95% CI [0.091, 0.189]); in the high-experience/low-mastery group, β = 0.125 (SE = 0.024, *p* < 0.001, 95% CI [0.084, 0.174]); and in the high-experience/high-mastery group, β = 0.234 (SE = 0.037, *p* < 0.001, 95% CI [0.168, 0.312]). A model allowing these indirect paths to vary across the four groups (χ²(152) = 201.3, *p* = 0.003) fit better than a model constraining them to be equal (χ²(164) = 234.8, *p* < 0.001; Δχ²(12) = 33.5, *p* = 0.001). We also estimated a continuous interaction model including both moderators simultaneously (AI Participation × Technological Experience and AI Literacy × Mastery Orientation). The three-way conditional indirect effect at high levels of both moderators (+1 SD) was β = 0.221 (SE = 0.034, *p* < 0.001, 95% CI [0.158, 0.291]), closely approximating the median-split estimate for the high-experience/high-mastery group (β = 0.234). Conversely, the conditional indirect effect at low levels of both moderators (−1 SD) was β = 0.067 (SE = 0.022, *p* = 0.002, 95% CI [0.027, 0.112]), consistent with the median-split low-experience/low-mastery estimate (β = 0.052). These convergent findings across analytical approaches support the robustness of the moderation patterns and suggest that the median-split results provide a reasonable approximation of the continuous moderation effects.

Moderation analysis revealed that the magnitude of the indirect pathway varied across learner profiles. For high-experience, mastery-oriented students, the sequential association from participation through literacy to critical thinking was strongest (β = 0.234, 95% CI [0.168, 0.312]): in this subgroup, greater AI participation was most strongly associated with subsequent AI literacy gains, and AI literacy was most strongly associated with subsequent critical thinking gains. Conversely, students with limited technological experience and performance orientation showed weaker indirect associations (β = 0.052, 95% CI [0.018, 0.095]), suggesting that the pathway from participation through literacy to critical thinking may be attenuated for learners who approach AI tools primarily as efficiency aids rather than learning opportunities. Notably, the direct association between AI Participation and Critical Thinking was non-significant for the performance-oriented group (*p* > 0.05), indicating that for these students, participation was primarily associated with critical thinking through its association with AI literacy development, rather than through direct mechanisms. These patterns highlight the importance of considering individual differences in designing AI-integrated curricula to promote equitable learning outcomes.

To address potential limitations associated with the categorization of continuous variables, we conducted a robustness check using Latent Moderated Structural Equations (LMS)^28,29^. This analysis estimated the interaction effects of Prior Technological Experience and Mastery Orientation on the structural paths without artificial grouping. Specifically, we estimated models including latent interaction terms: (a) AI Participation × Prior Technological Experience predicting AI Literacy, and (b) AI Literacy × Mastery Orientation predicting Critical Thinking. The LMS results corroborated the multi-group findings: the interaction term between AI Participation and Prior Technological Experience significantly predicted AI Literacy, and the interaction between AI Literacy and Mastery Orientation was similarly significant. Simple slope analyses confirmed that the association between participation and literacy strengthened as experience and mastery orientation increased (Supplementary Table S3)^30^. These continuous variable analyses confirm that the observed “Matthew Effect” is not an artifact of discretization.

### Model comparison and validation

Additional model comparison and sensitivity analyses are summarised in the Supplementary Material (Supplementary Table S2). We compared the proposed partial mediation model (M2; χ² = 145.3, df = 88, AIC = 15623.7, CFI = 0.951, RMSEA = 0.043) with a set of theoretically motivated alternatives, including no mediation (M1), complete mediation (M3), reverse causation (M4), bidirectional cross-lagged effects (M5), concurrent mediation (M6), and a multiple-mediator specification (M7). Models that removed the AI literacy pathway or imposed a reverse temporal ordering showed poorer fit and higher information criteria (e.g., M1 AIC = 15652.9; M4 AIC = 15667.8; M6 AIC = 15643.6). More complex extensions that added reciprocal paths or additional mediators yielded very similar global fit (M5 AIC = 15625.2; M7 AIC = 15626.1) and did not provide a statistically meaningful improvement over M2 based on the chi-square difference tests reported in Supplementary Table S2 (M5: Δχ² = 2.5, *p* = 0.287; M7: Δχ² = 5.8, *p* = 0.214). We therefore retain M2 as the primary specification on grounds of parsimony and a priori theoretical motivation. While model fit indices alone cannot definitively establish causal agency^24^, the temporal precedence established by the longitudinal design supports the theoretical plausibility of the hypothesized directional paths over reverse-causation alternatives. Accordingly, directional interpretations in this study are grounded primarily in the theoretical framework (Situated Learning Theory), the temporal ordering of measurements, and the a priori specification of hypotheses, rather than in post-hoc model comparisons alone. The cross-lagged panel design controls for prior levels of each construct and permits examination of whether associations are consistent with theoretical expectations, but cannot rule out unmeasured confounding or alternative causal processes.

Sensitivity analyses evaluated the robustness of the findings to alternative treatments of missing data, outliers, and model parameterisation. Analyses based on complete cases, analyses excluding univariate and multivariate outliers, and models with alternative sets of auxiliary variables yielded parameter estimates and confidence intervals that were highly similar to those of the primary FIML models. Finally, cross-validation procedures using random 60/40 splits of the sample showed that key standardized path coefficients and indirect effects were of comparable magnitude and statistical significance across subsamples, supporting the stability of the longitudinal patterns observed in the full dataset (Supplementary Table S2).

## Discussion

In this three-wave longitudinal study, higher levels of AI-assisted diagnosis participation were prospectively associated with higher medical critical thinking scores over a 12-month clinical rotation, after accounting for prior levels of all constructs and baseline covariates. These patterns do not support a simple “cognitive atrophy” view in which greater exposure to automated systems necessarily coincides with declines in human reasoning^7,8^. Instead, within a supervised clinical training context, students who were more actively involved in AI-assisted diagnosis tended to show stronger subsequent critical thinking. At the same time, these findings should not be interpreted as evidence that any particular AI system improves diagnostic reasoning. The design remains observational and relies on self-reported measures, and unmeasured factors such as general cognitive ability or conscientiousness may influence both AI participation and critical thinking. Rather than evaluating the effectiveness of a specific AI tool, the results speak to how AI augmented learning environments can be aligned with the development of analytic and reflective diagnostic practices when students are encouraged to engage critically with algorithmic recommendations^5,6,14^.

A central contribution of this analysis is the identification of AI literacy as a statistically mediating variable in the longitudinal association between students’ behavioural engagement with AI-assisted diagnosis (“participation”) and later critical thinking, thereby distinguishing technology usage from digital competence. Specifically, our model disentangles “participation” (behavioural frequency of interaction with AI-assisted diagnostic tasks) from “AI literacy” (the acquired competence to comprehend, evaluate, and appropriately integrate algorithmic outputs), addressing a common limitation in prior work that conflates use with competence. Drawing on Situated Learning Theory, the observed sequential pattern of associations is consistent with a developmental trajectory in which repeated, supervised participation (“learning by doing”) provides situated opportunities for students to actively acquire domain-relevant AI literacy^11^, which in turn is associated with more reflective and analytic reasoning and may serve as an intermediary competence linking participation behaviour to reasoning outcomes. Importantly, because these inferences are based on observational longitudinal mediation models, the indirect pathway should be interpreted as a decomposition of associations rather than definitive evidence of a causal mechanism; the results are consistent with, but do not prove, the theorised account. Future research using experimental or quasi-experimental variation in AI-supported training, objective performance measures, and richer confounder control is needed to test whether strengthening AI literacy causally amplifies the benefits of AI participation for clinical reasoning.

Students who understand the probabilistic nature of AI outputs are better equipped to interrogate diagnostic suggestions, identify algorithmic hallucinations, and synthesize discordant data^15^. Rather than treating AI literacy as a fixed prerequisite for using AI systems, it was conceptualised as a developing competence that can change during training. The cross lagged models indicated that higher participation was followed by higher AI literacy, and higher AI literacy was in turn associated with later gains in critical thinking, yielding a statistically significant indirect pathway across the three waves. This pattern is consistent with the idea that frequent, active participation in AI-assisted diagnosis can provide opportunities for students to learn how AI systems are built and evaluated, how to interpret uncertainty in algorithmic outputs, and how to integrate AI recommendations into their own diagnostic reasoning^5,11^. This finding clarifies that AI literacy is not merely a moderator that amplifies the effects of usage, but a developmental outcome of usage itself that subsequently enables higher-order reasoning. While theoretically plausible as a moderator, our explicit testing of temporal precedence via cross-lagged modelling confirms that AI literacy emerges from participation, structurally functioning as a mediator rather than a pre-existing boundary condition. The mediation analyses should nonetheless be viewed as evidence of an indirect longitudinal association rather than a definitive psychological mechanism. Future studies that combine performance-based AI literacy tasks, objective usage logs, and experimental or quasi-experimental designs will be needed to test whether targeted AI literacy interventions can causally strengthen the link between AI participation and critical thinking development.

The analysis of individual differences reveals that this developmental pathway is constrained by prior technological experience, reflecting a “Matthew Effect” in digital medical education where the “rich get richer.” Students with high prior experience derived significantly greater critical thinking benefits from AI participation compared to their less experienced peers. This aligns with Cognitive Load Theory, suggesting that novices may expend excessive cognitive resources merely navigating the interface and interpreting basic algorithmic outputs, leaving limited capacity for the metacognitive evaluation required for critical thinking^31^. Consequently, medical curricula cannot adopt a “one-size-fits-all” approach to AI integration. For students with limited technological backgrounds, foundational training in algorithmic logic must precede intensive clinical AI immersion to prevent cognitive overload and ensure equitable learning outcomes.

Furthermore, the moderating role of learning goal orientation—interpreted through Achievement Goal Theory—highlights the importance of student motivation in technology-mediated learning^17–19^. Mastery-oriented students, who view AI interactions as opportunities to understand the “why” behind a diagnosis, demonstrated the strongest mediation effects. In contrast, performance-oriented students, who may prioritize obtaining the “correct” diagnosis to validate competence, showed weaker critical thinking gains. This suggests that performance-oriented students may be more susceptible to “automation bias,” accepting AI outputs at face value to achieve rapid results^18,19^. To mitigate this, medical educators should restructure assessment strategies. Rather than evaluating students solely on diagnostic accuracy, assessments should require “explainable AI” tasks where students must explicitly justify their acceptance or rejection of an AI suggestion^20^. This pedagogical shift forces performance-oriented learners to engage with the reasoning process, potentially shifting their focus from outcome validation to competency development^9^. Although the statistical models treat AI literacy as a mediator rather than a moderator, the conditional pattern of associations should be interpreted as highlighting when and for whom AI participation is most likely to be linked with gains in critical thinking, rather than as evidence of a separate interaction effect.

Contextualizing these findings within the specific educational setting of the study enhances their transferability to global medical training. The participants were 4th and 5th-year students in the Chinese medical education system, engaging in full-time clinical rotations comparable to the “sub-internship” or early residency phases in North American and European models^32^. In this high-volume clinical environment, students frequently encounter diverse pathologies and are tasked with preliminary diagnostic workups under supervision. This intense “immersion” provides a unique ecological niche for AI integration, as the high case volume necessitates efficient tool use while offering ample iterations for “trial-and-error” learning with AI systems. Therefore, while the academic titles differ, the functional role of these students mirrors that of junior residents in other systems, suggesting that the observed benefits of AI participation on critical thinking are likely generalizable to postgraduate medical training contexts globally where clinical responsibility and AI tool availability intersect.

Based on these insights, we propose a multi-tiered framework for implementing AI in medical curricula, with supervision as the foundational prerequisite. First, stratified training is essential: novices require explicit instruction on AI limitations and prompt engineering before clinical application to reduce cognitive load, while advanced students should engage in “AI audit” exercises—deliberately identifying errors in AI diagnostic reports under faculty observation. Second, institutions must move beyond technical training to foster “epistemic agency,” teaching students to treat AI as a second opinion requiring validation rather than a gold standard for clinical judgment^2^. Third, and most critically, faculty development is paramount; clinical preceptors must be trained to model “skeptical usage” behaviours, explicitly verbalising their own critical evaluation of AI outputs during ward rounds and providing real-time feedback when students accept AI recommendations uncritically. This cultural shift, supported by institutional policy, ensures that AI remains a servant to clinical reasoning rather than its substitute. We emphasise that the observed benefits of AI participation on critical thinking likely depend on this supervisory infrastructure; implementing AI tools without concurrent investment in faculty training and structured oversight may fail to produce similar outcomes and could potentially undermine reasoning development.

Several limitations should be considered when interpreting these results. First, despite the longitudinal design and the use of cross-lagged panel models, the data remain observational and rely on self-reported measures of AI-assisted diagnosis participation, AI literacy, and medical critical thinking. As such, the analyses cannot establish causal effects of AI tools or AI literacy, and common method variance may inflate some of the associations observed. Relatedly, while the longitudinal design and cross-lagged panel models provide evidence of temporal precedence and statistical mediation, these analyses remain fundamentally correlational and cannot definitively establish that AI participation causally increases AI literacy, which in turn causally enhances critical thinking^24,25^. The interpretation of AI literacy as a mediating mechanism is grounded in theoretical reasoning from Situated Learning Theory and the temporal structure of the three-wave design, rather than in experimental manipulation or model fit comparisons that cannot adjudicate causal direction. Future research employing experimental or quasi-experimental designs—such as randomised controlled trials of AI literacy interventions—would provide stronger evidence for causal claims. This concern was partially addressed by modelling distinct constructs over three time points and testing longitudinal measurement invariance, but future research should incorporate objective indicators such as system usage logs, performance-based AI literacy tasks, and standardized assessments of diagnostic reasoning to triangulate the findings^10,25,33^. Second, the study was conducted in three universities within one national and cultural context using a specific AI-assisted diagnosis system embedded in high-volume teaching hospitals. Patterns of AI adoption, supervision norms, and digital infrastructure may differ in other countries, specialties, or institutional settings, which could influence both AI usage and educational outcomes^14^. Third, although key demographic and academic variables were controlled and several individual differences examined, other unmeasured factors—such as general cognitive ability, baseline clinical reasoning skills, faculty mentorship quality, and local curriculum design—may also shape how AI participation translates into AI literacy and critical thinking. Taken together, these limitations underscore the need for multi-site, multi-method studies that combine longitudinal designs with richer behavioural and performance data to better understand how AI-augmented learning environments influence the development of critical thinking in medical education.

Overall, this study contributes to ongoing debates in digital medicine and medical education about whether AI integration will erode or enhance key professional capacities. Across one year of supervised clinical training, more active participation in AI-assisted diagnosis was associated with gains in medical students’ critical thinking, partly through the pathway involving AI literacy development, and these associations were stronger among students with higher technological experience and mastery-oriented goals. These findings support viewing AI systems as learning resources that can scaffold, rather than replace, human diagnostic reasoning—but only when deployed within thoughtfully designed educational contexts that include structured faculty supervision, explicit AI literacy curricula, and assessment strategies that reward critical evaluation of algorithmic outputs. The benefits observed in this study should not be assumed to generalise to unsupervised settings. At the same time, they highlight that benefits are not automatic and can be unevenly distributed, reinforcing the importance of explicit AI literacy training, careful attention to individual differences, and institutional safeguards that keep clinical responsibility and critical judgment firmly in human hands^5,6,14,33^.

## Methods

### Study design and educational context

This investigation employed a prospective longitudinal cohort design over twelve months with three measurement waves: baseline (T₀), mid-point (T₁, 6 months), and follow-up (T₂, 12 months). The timing of the assessments was chosen to capture changes in medical critical thinking, which typically require extended periods of training to manifest measurable differences^31^. A one-month AI system familiarization period preceded baseline data collection so that students had sufficient exposure to the AI-assisted diagnosis tools before initial assessment.

The study was conducted across three medical universities in China. Participants were fourth- and fifth-year medical students engaged in full-time clinical rotations at large teaching hospitals. In the Chinese medical education system, these years involve intensive clinical immersion in which students perform preliminary patient workups, contribute to ward rounds, and work under close supervision. Functionally, this role is comparable to sub-interns or early postgraduate trainees in many international systems. During rotations, students had supervised access to hospital information systems and routinely used AI tools integrated into the clinical workflow^32^. Specifically, they engaged with (1) computer-aided diagnosis systems for radiological image interpretation (for example, detection of pulmonary nodules on CT scans) and (2) AI-driven clinical decision support systems embedded in the electronic health record that generate differential diagnosis suggestions based on structured symptom and examination data. This high-volume clinical environment ensured that AI-assisted diagnosis participation reflected authentic, practice-embedded interaction with diagnostic algorithms rather than simulated classroom exercises.

### Participants

A stratified cluster random sampling method was used to recruit participants, balancing representation across internal medicine, surgery, and comprehensive care rotations. Comprehensive power analysis employing G*Power 3.1.9 indicated *N* ≥ 420 required for detecting theoretically meaningful effect sizes (β ≥ 0.25) in longitudinal mediation models with 80% statistical power, α = 0.05, and anticipated attrition ≤20% across twelve-month intervals. The final analytical sample comprised 450 participants at baseline with 82.7% retention (*n* = 372) through final assessment, exceeding minimum requirements for stable parameter estimation in complex longitudinal models.

Attrition analysis indicated no significant differences in demographic characteristics or baseline levels of AI-assisted diagnosis participation, AI literacy, or medical critical thinking between retained and lost participants. Little’s MCAR test (χ² = 45.67, *p* = 0.32) supported the assumption that missingness was consistent with a Missing at Random mechanism. The mean age at baseline was 22.47 years (SD = 1.73), and 58.9% of the sample were women, reflecting the gender distribution of the regional medical student population. Comprehensive demographic characteristics and attrition statistics are reported in Table 5. Stratification considered institutional type (research-intensive vs teaching-focused), geographic distribution, and clinical specialization tracks to enhance external validity while maintaining representative sampling of the target population.

**Table 5:** Sample characteristics and demographic profile (*N* = 450)

Characteristic	Baseline sample (T₀)	Retained sample (T₂)	Attrition analysis
	*n* = 450	*n* = 372 (82.7%)	Statistical comparison
Demographic variables			
Age (years)			
Mean ± SD	22.47 ± 1.73	22.39 ± 1.69	*t*(448) = 0.84, *p* = 0.401
Range	20–26	20–26	*d* = 0.05
Gender, *n* (%)			
Female	263 (58.4)	219 (58.9)	χ²(1) = 0.11, *p* = 0.740
Male	187 (41.6)	153 (41.1)	φ = 0.02
Clinical year, n (%)			
Fourth year	201 (44.7)	167 (44.9)	χ²(1) = 0.02, *p* = 0.888
Fifth year	249 (55.3)	205 (55.1)	φ = 0.01
Academic performance indicators			
Cumulative GPA			
Mean ± SD	3.52 ± 0.39	3.54 ± 0.38	*t*(448) = −0.93, *p* = 0.354
Range	2.67–4.00	2.71–4.00	*d* = 0.05
Clinical rotation experience (months)			
Mean ± SD	9.34 ± 2.41	9.45 ± 2.38	*t*(448) = −0.78, *p* = 0.436
Range	5–16	5–16	*d* = 0.05
Institutional characteristics			
Institution type, *n* (%)			
Research-intensive	287 (63.8)	239 (64.2)	χ²(1) = 0.09, *p* = 0.764
Teaching-focused	163 (36.2)	133 (35.8)	φ = 0.01
Geographic distribution, *n* (%)			
Urban	298 (66.2)	248 (66.7)	χ²(1) = 0.12, *p* = 0.729
Peri-urban	152 (33.8)	124 (33.3)	φ = 0.02
Specialization track, *n* (%)			
Internal medicine	167 (37.1)	139 (37.4)	χ²(2) = 0.23, *p* = 0.891
Surgery	152 (33.8)	125 (33.6)	Cramer’s V = 0.02
Comprehensive care	131 (29.1)	108 (29.0)	
Baseline technology experience			
Prior AI system exposure, *n* (%)			
None	167 (37.1)	134 (36.0)	χ²(2) = 1.12, *p* = 0.571
Limited (1–6 months)	198 (44.0)	168 (45.2)	Cramer’s V = 0.05
Moderate (7+ months)	85 (18.9)	70 (18.8)	
Technology proficiency level			
Mean ± SD	4.31 ± 1.22	4.36 ± 1.19	*t*(448) = −0.71, *p* = 0.478
Range	1–7	1–7	*d* = 0.04
Medical technology familiarity			
Mean ± SD	3.89 ± 1.34	3.94 ± 1.31	*t*(448) = −0.67, *p* = 0.503
Range	1–7	1–7	*d* = 0.04
Individual difference measures			
Prior technological experience scale			
Mean ± SD	4.18 ± 1.28	4.22 ± 1.25	*t*(448) = −0.58, *p* = 0.562
Range	1–7	1–7	*d* = 0.03
Learning goal orientation			
Mastery orientation (mean ± SD)	5.78 ± 0.91	5.81 ± 0.89	*t*(448) = −0.62, *p* = 0.535
Performance orientation (mean ± SD)	4.12 ± 1.43	4.09 ± 1.41	*t*(448) = 0.39, *p* = 0.697

Comprehensive attrition analysis indicates no significant demographic differences between retained and lost participants across all measured variables, supporting missing-at-random assumptions for subsequent analytical procedures. Effect sizes: *d* = Cohen’s *d* for continuous variables; φ = phi coefficient for 2 × 2 contingency tables; Cramer’s V for larger contingency tables. Technology proficiency and familiarity assessed on 7-point Likert scales (1 = very low proficiency/familiarity, 7 = very high proficiency/familiarity). Learning goal orientations assessed using validated psychometric instruments with established reliability and validity evidence. Missing data for any variable ≤ 1.8% across all assessment domains. Statistical power for attrition analyses ranged from 0.82 to 0.95 for detecting medium effect sizes (*d* ≥ 0.30) at α = 0.05, ensuring adequate sensitivity for identifying systematic attrition patterns.

### Measures

All instruments employed 7-point Likert response scales to optimise measurement precision and distributional variability. To mitigate threats from common method variance (CMV), we implemented a multi-faceted methodological strategy encompassing both procedural and statistical safeguards. Procedurally, temporal separation across the three measurement waves reduced consistency motifs and retrospective recall biases, while psychological separation techniques—including varied response anchors, counterbalanced item sequencing, and the insertion of methodologically unrelated buffer items—diminished respondent hypothesis-guessing and socially desirable responding. Conceptually, we maintained strict theoretical and operational differentiation between AI-assisted diagnosis participation (operationalised as behavioural engagement frequency and depth) and AI literacy (operationalised as cognitive competencies in algorithmic comprehension and evaluation), ensuring minimal construct conflation. Statistically, Harman’s single-factor test indicated that no single factor accounted for the majority of covariance (single-factor variance explained = 31.2%), and a confirmatory single-factor model demonstrated substantially degraded fit (CFI = 0.67, RMSEA = 0.14) relative to the hypothesised multi-factor measurement model, collectively suggesting that CMV does not pose a serious threat to the validity of the observed associations.

### AI-assisted diagnosis participation (behaviour)

The AI-Assisted Diagnosis Participation Scale (AIDPS-15) represents a comprehensive measure integrating behavioural frequency, cognitive engagement depth, and systematic utilization dimensions based on established technology acceptance and engagement frameworks. The 15-item instrument employs 7-point Likert scaling with behavioural anchoring, incorporating items assessing participation frequency (“How often do you actively engage with AI diagnostic systems during clinical activities?”), analytical depth (“To what extent do you critically evaluate AI diagnostic recommendations before clinical application?”), and systematic integration (“How frequently do you combine AI insights with clinical reasoning in diagnostic decision-making?”). Items were rated from 1 (“never/strongly disagree”) to 7 (“very often/strongly agree”), with higher scores indicating more active and reflective participation in AI-assisted diagnosis.

The scale showed excellent internal consistency across waves (Cronbach’s α = 0.89–0.93) and good temporal stability (test–retest *r* = 0.81 between adjacent waves). Confirmatory factor analysis supported a three-factor structure corresponding to the frequency, depth, and integration dimensions. Convergent validity was indicated by moderate correlations with faculty ratings of student AI engagement (*r* = 0.64) and peer assessments of technological participation (*r* = 0.58). Discriminant validity was supported by weaker correlations with general technology enthusiasm measures (*r* = 0.23), suggesting that the scale captures participation in clinical AI use rather than general ICT liking.

### AI literacy (competence)

AI literacy was conceptualised as a competence distinct from usage behaviour. It was measured using an adapted version of the Medical Artificial Intelligence Readiness Scale^12^, aligned with core AI literacy components described by Long and Magerko^11^. Items assessed students’ perceived ability to understand, evaluate, and work with AI outputs in clinical contexts. Example items include “I can identify potential biases in the data used by the AI model”, “I understand the limitations of the AI system’s probabilistic reasoning”, and “I can explain why an AI recommendation may not be appropriate for a specific patient”. Responses were given on a 7-point scale from 1 (“strongly disagree”) to 7 (“strongly agree”).

Internal consistency of the adapted AI literacy scale was high across waves (α ≥ 0.90). To address concerns about construct overlap with AI-assisted diagnosis participation, we conducted a discriminant validity analysis. The square root of the Average Variance Extracted (AVE) for AI literacy (0.84) exceeded its correlation with AI-assisted diagnosis participation (*r* = 0.64), providing statistical evidence that the two constructs are related but distinct (behaviour vs competence).

### Medical critical thinking

Medical critical thinking was assessed using the Medical Critical Thinking Scale–Revised (MCTS-R-18), a domain-adapted version of established critical thinking assessments grounded in Facione’s critical thinking taxonomy^13^. The scale comprises 18 items capturing analytical reasoning (for example, “I systematically evaluate the evidence before reaching diagnostic conclusions”), inference (for example, “I can identify logical relationships between clinical findings”), and metacognitive regulation (for example, “I monitor my reasoning processes during diagnostic decision-making”). Items were rated from 1 (“strongly disagree”) to 7 (“strongly agree”), with higher scores indicating stronger critical thinking dispositions in medical contexts.

The MCTS-R-18 demonstrated good internal consistency across waves (α = 0.87–0.91). Previous research has supported its convergent and discriminant validity in medical education contexts, and in the present sample, item distributions and factor loadings were consistent with prior validation studies.

### Individual difference measures

Two baseline individual difference variables were assessed as potential moderators of the longitudinal associations: prior technological experience and learning goal orientation. Prior technological experience was measured using a 6-item scale assessing baseline competence with medical information systems, previous exposure to AI tools in clinical or educational settings, and general ICT proficiency. Items were rated on a 7-point scale, and higher scores indicated more extensive and confident use of technology.

Learning goal orientation was measured using an 8-item instrument adapted from Dweck and Leggett’s achievement goal framework. Items distinguished mastery orientation (for example, “I prefer challenging learning opportunities that help me develop diagnostic skills”) from performance orientation (for example, “I prefer clinical situations where I can demonstrate my competence to supervisors”). The instrument employed forced-choice wording to reduce social desirability bias. In this study, mastery and performance orientation scores were treated as continuous indices. Both individual difference measures showed acceptable reliability (α ≥ 0.83) and temporal stability, with test–retest correlations ranging from 0.76 to 0.82 across measurement occasions. Detailed item content and additional psychometric information for all scales are provided in the Supplementary Table S1. These individual difference measures were administered at all three waves to examine temporal stability; however, only T₀ scores were used as time-invariant moderators in the longitudinal structural models.

### Procedure

Ethical approval for this study was obtained from the Ethics Committee of Guangxi Orthopaedic Hospital (Approval No. 20221212). All procedures were conducted in accordance with the Declaration of Helsinki and local institutional guidelines. Students were informed about the study aims and procedures during scheduled teaching sessions and were invited to participate voluntarily. Written informed consent was obtained from all participants before data collection.

Questionnaires were administered online via a secure institutional survey platform at each of the three time points. To minimise social desirability bias and perceived coercion, students were assured that their responses would remain confidential, would not be shared with clinical supervisors in an identifiable form, and would have no impact on course grades or clinical performance evaluations. Participants could withdraw from the study at any time without penalty.

### Data analysis

Data analyses were conducted using Mplus version 8.4. Reporting of this observational cohort study follows the STROBE guidelines for longitudinal observational research. Descriptive statistics and bivariate correlations were calculated for all variables at each wave. Confirmatory factor analyses were conducted to evaluate the measurement structure of the multi-item scales, and longitudinal measurement invariance (configural, metric, and scalar) was tested to ensure that construct scores were comparable over time.

The primary analysis tested a three-wave cross-lagged panel mediation model in which AI-assisted diagnosis participation at each wave predicted subsequent AI literacy and medical critical thinking, while controlling for stability paths and concurrent associations among constructs^34^. Baseline age, gender, and grade point average (GPA) were included as covariates predicting the endogenous variables to reduce confounding. Longitudinal mediation was examined by estimating indirect paths from baseline AI-assisted diagnosis participation (T₀) to critical thinking at T₂ via AI literacy at T₁.

To handle missing data arising from attrition and item non-response, we used Full Information Maximum Likelihood (FIML) estimation under a Missing at Random assumption^35,36^. The inclusion of baseline demographic and academic variables as auxiliary variables in the FIML estimation was intended to improve the plausibility of this assumption and reduce bias associated with selective attrition, auxiliary variables included age, gender, institution type, baseline GPA, and baseline levels of AI participation, AI literacy, and medical critical thinking^35,36^. Item-level missingness was below commonly recommended thresholds for FIML in longitudinal structural equation modelling.

Primary mediation analyses employed Monte Carlo confidence intervals with 10,000 resamples to estimate the precision of indirect effects. To examine whether the indirect pathways differed across learner profiles, we conducted multi-group analyses in which the sample was divided by median splits on prior technological experience, mastery orientation, and performance orientation^37^. In these secondary analyses, equality constraints on the key structural paths were compared with unconstrained models using χ² difference tests to evaluate moderation. As a robustness check, sensitivity analyses treating moderators as continuous variables were also conducted; these yielded similar patterns of effects and are reported in the Supplementary Material.

Model fit was evaluated using multiple indices: χ² statistics, the Comparative Fit Index (CFI), Tucker–Lewis Index (TLI), Root Mean Square Error of Approximation (RMSEA), and Standardized Root Mean Square Residual (SRMR). CFI and TLI values ≥ 0.95, RMSEA ≤ 0.06, and SRMR ≤ 0.08 were considered indicative of good fit, while recognising that rigid adherence to fixed cutoffs is not recommended and that model evaluation should consider the pattern of indices, sample size, and model complexity^34,36,37^.

Methodologically, we employed both median-split and continuous-variable approaches to examine moderation effects. The primary analyses used median splits to facilitate clinical interpretability and to identify distinct learner profiles that may benefit from differentiated instructional approaches. However, acknowledging concerns about statistical power loss and arbitrary boundary effects associated with dichotomisation^38^, we conducted supplementary latent moderated structural equations analyses treating all moderators as continuous variables. The convergence of findings across these complementary approaches—with continuous-variable interaction effects reaching significance and conditional indirect effects closely approximating median-split estimates—strengthens confidence in the robustness of the moderation patterns observed. The median-split presentations in the main text serve pedagogical and practical purposes, while the continuous-variable results in Supplementary Table S3 provide more statistically rigorous estimates for methodologically oriented readers.

## Supplementary information

Supplementary Information

## Data Availability

The datasets generated and analysed during the current study are not publicly available due to institutional data protection regulations, but de-identified data may be made available from the corresponding author on reasonable request and with approval from the relevant institutional bodies.

## Code availability

All custom code used in this study is available from the corresponding author upon reasonable request. The longitudinal structural equation models were estimated in Mplus (Muthén & Muthén) using robust maximum likelihood estimation with full information maximum likelihood (FIML) for handling missing data. Descriptive and supplementary analyses were conducted in R (R Foundation for Statistical Computing). The Mplus syntax files and R scripts specify all variables, model parameters, and options used to generate and analyse the current datasets.
