# Supplementary material for: AI literacy mediates AI assisted diagnosis participation and critical thinking among medical students under supervision
Source: NPJ Digit Med. 2026 Mar 14;9:344. doi: 10.1038/s41746-026-02521-9 (PMC13133384; doi:10.1038/s41746-026-02521-9)
Supplement: Supplementary file 1 — Supplementary Information [file 41746_2026_2521_MOESM1_ESM.pdf]

Supplementary Material Table S1

Descriptive Statistics and Psychometric Properties of Primary Measures (N = 372)

Measure	Time Point	M	SD	Range	Skewness	Kurtosis	α	ω	Test-Retest r
**AI-Assisted Diagnosis Participation (AIDPS-15)**									
Total Score	T_0 (Baseline)	3.12	1.18	1.00-6.87	0.34	-0.41	.89	.91	.81
	T_1 (6 months)	4.67	1.09	1.93-7.00	-0.22	-0.38	.91	.93	.84
	T_2 (12 months)	5.41	0.98	2.47-7.00	-0.45	-0.15	.93	.94	.86
Behavioral Frequency	T_0	3.24	1.31	1.00-7.00	0.28	-0.52	.84	.86	.78
	T_1	4.89	1.15	1.40-7.00	-0.31	-0.29	.86	.88	.80
	T_2	5.67	1.02	2.80-7.00	-0.53	0.12	.88	.90	.82
Cognitive Depth	T_0	2.89	1.25	1.00-6.60	0.41	-0.33	.82	.84	.76
	T_1	4.51	1.18	1.80-7.00	-0.18	-0.44	.84	.86	.78
	T_2	5.23	1.08	2.20-7.00	-0.38	-0.22	.86	.88	.80
Systematic Integration	T_0	3.23	1.34	1.00-6.80	0.32	-0.48	.85	.87	.79
	T_1	4.62	1.21	1.60-7.00	-0.17	-0.41	.87	.89	.81
	T_2	5.34	1.11	2.40-7.00	-0.43	-0.18	.89	.91	.83
**AI Literacy**									
Total Score	T_0 (Baseline)	3.45	0.87	1.44-6.22	0.15	-0.28	.90	.92	.79
	T_1 (6 months)	4.89	0.79	2.39-6.78	-0.24	-0.21	.92	.94	.82
	T_2 (12 months)	5.67	0.73	3.11-7.00	-0.38	0.19	.94	.96	.85
Technical Proficiency	T_0	3.67	0.94	1.60-6.40	0.09	-0.35	.87	.89	.76
	T_1	5.12	0.85	2.80-6.80	-0.31	-0.18	.89	.91	.79
	T_2	5.89	0.76	3.40-7.00	-0.45	0.22	.91	.93	.82
Information Evaluation	T_0	3.28	0.96	1.20-6.00	0.21	-0.31	.85	.87	.74
	T_1	4.71	0.88	2.20-6.60	-0.19	-0.25	.87	.89	.77
	T_2	5.51	0.81	3.00-7.00	-0.33	0.18	.89	.91	.80
Integration Capabilities	T_0	3.41	0.99	1.40-6.20	0.17	-0.29	.83	.85	.72
	T_1	4.84	0.91	2.40-6.80	-0.22	-0.23	.85	.87	.75
	T_2	5.61	0.84	3.20-7.00	-0.36	0.16	.87	.89	.78
**Critical Thinking (MCTS-R-18)**									
Total Score	T_0 (Baseline)	4.23	0.68	2.44-6.11	-0.08	-0.39	.87	.89	.83
	T_1 (6 months)	4.52	0.65	2.78-6.22	-0.19	-0.32	.89	.91	.85
	T_2 (12 months)	4.78	0.64	3.06-6.33	-0.27	-0.21	.91	.93	.87
Analytical Reasoning	T_0	4.31	0.74	2.20-6.20	-0.12	-0.41	.84	.86	.80
	T_1	4.61	0.71	2.60-6.40	-0.23	-0.29	.86	.88	.82

Measure	Time Point	M	SD	Range	Skewness	Kurtosis	α	ω	Test-Retest r
	T_2	4.89	0.69	3.20-6.40	-0.31	-0.18	.88	.90	.84
Inference Capabilities	T_0	4.18	0.79	2.40-6.00	-0.05	-0.38	.82	.84	.78
	T_1	4.45	0.76	2.80-6.20	-0.17	-0.35	.84	.86	.80
	T_2	4.71	0.73	3.00-6.20	-0.25	-0.22	.86	.88	.82
Metacognitive Regulation	T_0	4.21	0.82	2.60-6.20	-0.07	-0.42	.80	.82	.76
	T_1	4.51	0.78	2.80-6.40	-0.18	-0.36	.82	.84	.78
	T_2	4.75	0.76	3.20-6.40	-0.26	-0.20	.84	.86	.80

Note. All measures employ 7-point Likert scaling (1 = strongly disagree, 7 = strongly agree). α = Cronbach's alpha internal consistency reliability; ω = McDonald's omega coefficient; Test-Retest r = temporal stability coefficient across 4-month intervals. All reliability coefficients exceed established .80 threshold for research applications. Distributional characteristics support assumptions for latent variable modeling procedures. Progressive improvement across temporal intervals validates theoretical predictions regarding technology-mediated competency development trajectories.

Supplementary Material Table S2

Model Comparison and Sensitivity Analysis (N = 372)

Model Specification	χ^2	df	AIC	BIC	CFI	RMSEA	$\Delta\chi^2$	p
**Theoretical Model Comparisons**								
M1: No Mediation	178.6	90	15652.9	15789.4	.938	.051	—	—
M2: Partial Mediation (Proposed)	145.3	88	15623.7	15767.8	.951	.043	33.3	<.001
M3: Complete Mediation	154.0	89	15630.4	15771.7	.947	.045	8.7	.003
M4: Reverse Causation	189.4	88	15667.8	15811.9	.932	.056	44.1	<.001
M5: Bidirectional	142.8	86	15625.2	15776.9	.953	.042	2.5	.287
**Alternative Specifications**								
M6: Concurrent Mediation	167.2	89	15643.6	15784.9	.944	.049	21.9	<.001
M7: Multiple Mediators	139.5	84	15626.1	15784.4	.955	.041	5.8	.214
**Preferred Model: M2 (Partial Mediation)**								
**Sensitivity Analysis Results**								
**Missing Data Treatment**	**Effect**	**95% CI**	**Interpretation**					
FIML (Original)	β = .142, p < .001	[.089, .201]	Baseline result					
Multiple Imputation (m=20)	β = .138, p < .001	[.086, .195]	Minimal bias					
Complete Cases Only	β = .147, p < .001	[.091, .208]	Robust effect					
**Outlier Treatment**								
All Cases Included	β = .142, p < .001	[.089, .201]	Baseline result					
Winsorized (5%)	β = .139, p < .001	[.087, .196]	Stable effect					
Outliers Removed (n=12)	β = .144, p < .001	[.090, .203]	Robust findings					
**Alternative Timeframes**								
Standard (6, 12 months)	β = .142, p < .001	[.089, .201]	Baseline result					
Compressed (3, 9 months)	β = .118, p = .002	[.067, .174]	Reduced effect					
Extended (9, 15 months)	β = .156, p < .001	[.098, .219]	Enhanced effect					
**Cross-Validation**								
Estimation Sample (60%, n=223)	β = .149, p < .001	[.084, .223]	Consistent effect					
Validation Sample (40%, n=149)	β = .134, p = .003	[.071, .208]	Replicated findings					
**Effect Size Benchmarking**								
**Reference Domain**	**Typical Range**	**Current Study**	**Comparison**					
Educational Technology Meta-Analysis	β = .10-.20	β = .142	Within range					

Model Specification	χ^2	df	AIC	BIC	CFI	RMSEA	$\Delta\chi^2$	p
Medical Education Interventions	β = .12-.18	β = .142	Above average					
Longitudinal Mediation Studies	β = .08-.15	β = .142	Upper range					
**Practical Significance Thresholds**								
Minimum Detectable Effect	β ≥ .10	✓ Exceeded						
Educational Importance	β ≥ .12	✓ Exceeded						
Policy Relevance	β ≥ .15	≈ Approached						

Note. Model comparison employs nested chi-square difference tests for theoretical model evaluation. FIML = Full Information Maximum Likelihood estimation. Sensitivity analyses demonstrate robustness across analytical assumptions and methodological variations. Effect size benchmarking draws from established meta-analyses in educational technology and medical education research domains. Cross-validation procedures support external validity within study population parameters. Practical significance thresholds based on established educational research conventions and policy implementation requirements.

Supplementary Material Table S3

Continuous-Variable Moderation Analysis Using Latent Moderated Structural Equations (N = 372)

Panel A: Latent Interaction Effects

Effect	β	SE	z	p	95% CI
**Model 1: Technological Experience as Moderator**					
**Main Effects**					
AI Participation T_0 → AI Literacy T_1	.327	.038	8.61	<.001	[.253, .401]
Prior Tech Experience → AI Literacy T_1	.198	.042	4.71	<.001	[.116, .280]
**Interaction Effect**					
AI Participation × Tech Experience → AI Lit T_1	.089	.031	2.87	.004	[.028, .150]
Model Fit: $\chi^2(94) = 156.3$, CFI = .948, RMSEA = .042, SRMR = .039					
**Model 2: Mastery Orientation as Moderator**					
**Main Effects**					
AI Literacy T_1 → Critical Thinking T_2	.274	.033	8.30	<.001	[.209, .339]
Mastery Orientation → Critical Thinking T_2	.187	.039	4.79	<.001	[.111, .263]
**Interaction Effect**					
AI Literacy × Mastery Orient → Crit Think T_2	.076	.028	2.71	.007	[.021, .131]
Model Fit: $\chi^2(94) = 152.7$, CFI = .951, RMSEA = .041, SRMR = .038					
**Model 3: Combined Moderation (Both Moderators Simultaneously)**					
**Interaction Effects**					
AI Participation × Tech Experience → AI Lit T_1	.084	.032	2.63	.009	[.021, .147]
AI Literacy × Mastery Orient → Crit Think T_2	.071	.029	2.45	.014	[.014, .128]
Model Fit: $\chi^2(142) = 198.4$, CFI = .946, RMSEA = .043, SRMR = .041					

Panel B: Simple Slope Analysis

Moderator Level	Simple Slope (β)	SE	z	p	95% CI
**AI Participation → AI Literacy (Moderated by Tech Experience)**					
Low Tech Experience (−1 SD)	.238	.047	5.06	<.001	[.146, .330]
Mean Tech Experience	.327	.038	8.61	<.001	[.253, .401]
High Tech Experience (+1 SD)	.416	.049	8.49	<.001	[.320, .512]
Slope Difference (High − Low)	.178	.062	2.87	.004	[.056, .300]
**AI Literacy → Critical Thinking (Moderated by Mastery Orientation)**					
Low Mastery Orientation (−1 SD)	.205	.044	4.66	<.001	[.119, .291]
Mean Mastery Orientation	.274	.033	8.30	<.001	[.209, .339]
High Mastery Orientation (+1 SD)	.343	.046	7.46	<.001	[.253, .433]
Slope Difference (High − Low)	.138	.051	2.71	.007	[.038, .238]

Panel C: Conditional Indirect Effects

Moderator Configuration	Indirect Effect	SE	p	95% CI
**Single Moderator Models**				
**Prior Technological Experience**				
Low (−1 SD)	.089	.025	<.001	[.043, .141]
Mean	.122	.021	<.001	[.083, .165]
High (+1 SD)	.162	.029	<.001	[.109, .222]
**Mastery Orientation**				
Low (−1 SD)	.095	.024	<.001	[.051, .145]
Mean	.127	.020	<.001	[.089, .168]
High (+1 SD)	.168	.027	<.001	[.118, .224]
**Combined Moderator Model (Both Moderators)**				
Low Tech Exp (−1 SD) × Low Mastery (−1 SD)	.067	.022	.002	[.027, .112]
Low Tech Exp (−1 SD) × High Mastery (+1 SD)	.124	.028	<.001	[.073, .181]
High Tech Exp (+1 SD) × Low Mastery (−1 SD)	.118	.027	<.001	[.069, .174]
High Tech Exp (+1 SD) × High Mastery (+1 SD)	.221	.034	<.001	[.158, .291]
**Index of Moderated Mediation**				
Tech Experience	.037	.014	.008	[.010, .065]
Mastery Orientation	.041	.016	.010	[.011, .074]

Panel D: Comparison with Median-Split Results

Moderator Configuration	Continuous Model	Median-Split	Δβ	Convergence
**Tech Experience**				
Low	.089	.096	−.007	Excellent
High	.162	.178	−.016	Excellent
**Mastery Orientation**				
Low	.095	.081	+.014	Excellent
High	.168	.189	−.021	Good
**Combined**				
Low × Low	.067	.052	+.015	Good
High × High	.221	.234	−.013	Excellent
Overall Correlation	r = .987			Convergent

Note. All analyses employed latent moderated structural equations (LMS) estimation with robust maximum likelihood. Interaction effects were estimated using the product indicator approach with mean-centered indicators. Simple slopes computed at ±1 SD of moderator variables. Conditional indirect effects computed using Monte Carlo simulation with 10,000 resamples. Index of moderated mediation indicates the rate of change in the indirect effect per one-unit increase in the moderator. Panel D demonstrates convergence between continuous-variable and median-split approaches; differences are attributable to information loss in dichotomisation and boundary effects at the median cut-point. Tech Exp = Prior Technological Experience; Mastery = Mastery Goal Orientation. β = standardised coefficient; SE = standard error; CI = confidence interval.
